# Profiling Cellular Processes in Adipose Tissue during Weight Loss Using Time Series Gene Expression

**DOI:** 10.3390/genes9110525

**Published:** 2018-10-29

**Authors:** Samar H. K. Tareen, Michiel E. Adriaens, Ilja C. W. Arts, Theo M. de Kok, Roel G. Vink, Nadia J. T. Roumans, Marleen A. van Baak, Edwin C. M. Mariman, Chris T. Evelo, Martina Kutmon

**Affiliations:** 1 Maastricht Centre for Systems Biology (MaCSBio), Maastricht University, 6211ER Maastricht, The Netherlands; ilja.arts@maastrichtuniversity.nl (I.C.W.A.); t.dekok@maastrichtuniversity.nl (T.M.d.K.); 2 Department of Epidemiology, CARIM School for Cardiovascular Diseases, Maastricht University, 6211ER Maastricht, The Netherlands; 3 Department of Toxicogenomics, GROW School of Oncology and Developmental Biology, Maastricht University, 6211ER Maastricht, The Netherlands; 4 Department of Human Biology, NUTRIM Research School, Maastricht University, 6211ER Maastricht, The Netherlands; r.vink@maastrichtuniversity.nl (R.G.V.); n.roumans@maastrichtuniversity.nl (N.J.T.R.); m.vanbaak@maastrichtuniversity.nl (M.A.v.B.); e.mariman@maastrichtuniversity.nl (E.C.M.M.); 5 Department of Bioinformatics—BiGCaT, NUTRIM Research School, Maastricht University, 6211ER Maastricht, The Netherlands

**Keywords:** obesity, diet, adipose tissue, correlation networks, transcriptomics, differential expression, cellular processes, cytoscape, network biology, network visualisation

## Abstract

Obesity is a global epidemic identified as a major risk factor for multiple chronic diseases and, consequently, diet-induced weight loss is used to counter obesity. The adipose tissue is the primary tissue affected in diet-induced weight loss, yet the underlying molecular mechanisms and changes are not completely deciphered. In this study, we present a network biology analysis workflow which enables the profiling of the cellular processes affected by weight loss in the subcutaneous adipose tissue. Time series gene expression data from a dietary intervention dataset with two diets was analysed. Differentially expressed genes were used to generate co-expression networks using a method that capitalises on the repeat measurements in the data and finds correlations between gene expression changes over time. Using the network analysis tool Cytoscape, an overlap network of conserved components in the co-expression networks was constructed, clustered on topology to find densely correlated genes, and analysed using Gene Ontology enrichment analysis. We found five clusters involved in key metabolic processes, but also adipose tissue development and tissue remodelling processes were enriched. In conclusion, we present a flexible network biology workflow for finding important processes and relevant genes associated with weight loss, using a time series co-expression network approach that is robust towards the high inter-individual variation in humans.

## 1. Introduction

In recent years, obesity has become a global epidemic with a World Health Organisation (WHO) estimate of 11% men and 15% women worldwide being obese in 2014 [1], with clear indications that this will continue to rise in the foreseeable future. Obesity has been identified as a major risk factor for multiple diseases and conditions such as type 2 diabetes mellitus (T2DM), cardiovascular diseases (CVD) and the metabolic syndrome (MetS) [2,3,4]. Consequently, a number of studies [5,6,7] have recommended weight loss through diet and physical activity to counter obesity and its co-morbidities.

In previous studies, various efforts have been made to understand the molecular biology behind obesity and the effect of weight loss [8,9,10]. These studies have focused on gene expression profiles and targeted pathways associated with the obese system, providing transcriptomic snapshots to better understand the functioning of the system in a particular state or in responses to stimuli by comparing the expression of the genes across the genome.

In recent years, gene expression profiling has been extended into time series, generating gene expression snapshots at different time points. The time points collectively show how gene expression changes over time, particularly in response to different stimuli or interventions. The time series also yield differential gene expression data which provides expression contrasts between pairs of time points, which are used to glean which processes may or may not be active at a particular time point via cellular pathways [11]. However, it has been difficult to accurately associate cellular processes and pathways with time series gene expression profiles, partly due to high inter-individual variability in human transcriptomic datasets [12,13].

In this article, we present a network biology analysis workflow which ties time series gene expression analysis with gene expression pattern correlation and downstream enrichment techniques to identify biological processes and pathways in the adipose tissue as possible regulatory candidates linking the obese system and chronic diseases with the beneficial effects of weight loss. The analyses give us a detailed view of what is happening in the subcutaneous adipose tissue during weight loss, irrespective of the intensity and duration of caloric restriction. Furthermore, the analysis provides us with closely correlated clusters of genes based on their gene expression patterns. These clusters represent possible areas of cross-talk between different biological processes, increasing our understanding of the functioning of the subcutaneous adipose tissue, as well as providing us with new areas of detailed research in the context of obesity and chronic diseases. In addition, the described network biology workflow is able to perform these analyses on human datasets containing high variation between the participants of the study, allowing for application on small sized studies as well.

## 2. Materials and Methods

### 2.1. Analysis Workflow

**Figure 1:**
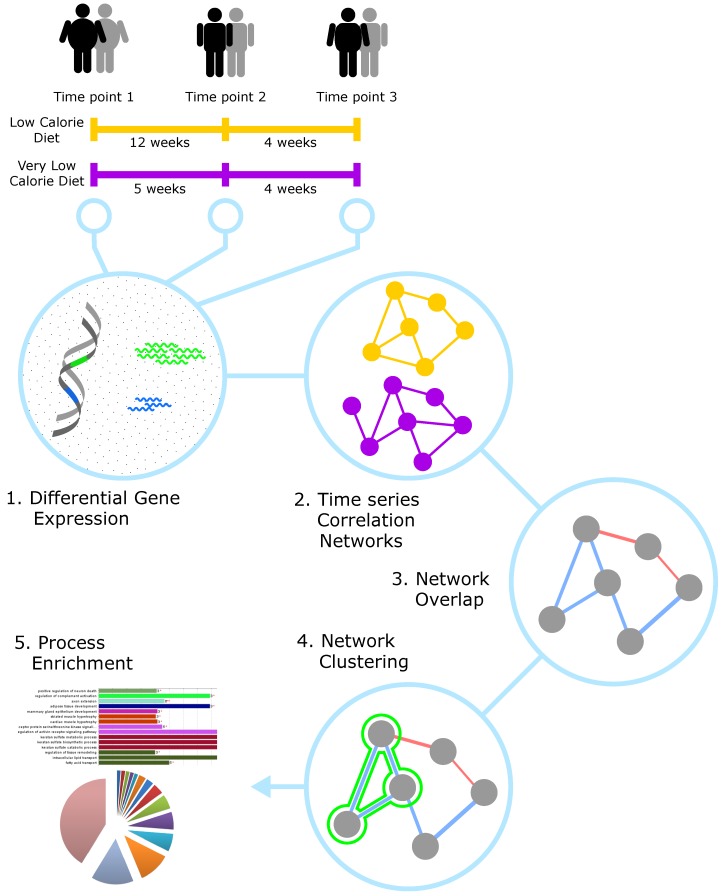
Network biology analysis workflow. (**1**) The time series expression data from the time points is normalised and differential expression analysis is performed. (**2**) Correlation networks are constructed on the time series for each diet respectively. (**3**) The overlap network is generated from the two networks showing the correlations which are shared between the two diets. (**4**) Community clustering is performed to find clusters of genes which are showing the most similar expression patterns. (**5**) The overlap network and the gene clusters are then used for process enrichment to find the affected cellular processes.

The steps in our network biology analysis workflow is shown in Figure 1 and explained in detail in the following subsections. This workflow follows a data driven approach, taking raw expression data from microarrays to computable networks and information relating to biological processes. The data pre-processing (step 1) and constructing of correlation networks (step 2) are performed in ArrayAnalysis [14] and R [15]. The network analysis steps (3–5) are performed in Cytoscape [16] using core and app functionality.

### 2.2. Dataset

Raw transcriptomics data was obtained from the ‘Yoyo study’ [17] (Clinical Trial ID: NCT01559415, www.clinicaltrials.gov). The study was a human weight loss and subsequent weight regain study, comparing two weight loss diets: a low calorie diet (LCD) of 1250 kcal/day for 12 weeks, and a very low calorie diet (VLCD) of 500 kcal/day for five weeks. Participants of each diet then underwent a four week weight maintenance period, with a nine-month follow up. All participants in the study were overweight and/or obese Caucasian individuals with body mass index (BMI) from 28 kg/m${}^{2}$ to 35 kg/m${}^{2}$ aged from 32 to 67 years (median age 51). The exact details of the study design can be found in [17].

The transcriptomics data was available on the Gene Expression Omnibus (ID: GSE77962). The data consisted of Affymetrix Human Gene ST 1.1 microarray platform expression data of the subcutaneous adipose tissue for 57 individuals. The complete gene expression data for the first three time points of the study (before weight loss, after weight loss, after weight maintenance) was available for only 46 subjects (22 LCD and 24 VLCD). The transcriptomics data was not available for the final time point of the study.

### 2.3. Data Quality Control and Filtration of Background Expression

ArrayAnalysis.org was used for the quality control (QC) analysis and subsequent normalisation of the raw expression data [14]. ArrayAnalysis.org is an online pipeline for QC and normalisation of microarray expression data. In our study, we used the default settings for Affymetrix Human Gene ST 1.1 microarray platform and normalised using robust multi-array averaging (RMA) normalisation. The normalised expression data was then filtered for noise, generated via background gene expression, by removing genes which had median expression in the dataset equal-to or lower-than the median Y-chromosome gene expressions in female individuals in the dataset.

### 2.4. Differential Expression Analysis

The filtered expression data was then used for differential expression analysis of the genes across the three time points, separately for each diet. This analysis allowed us to compare how the gene expressions changed after weight loss and after weight maintenance. Comparing the number of significantly differentially expressed genes using the criteria |FC| ≥ 1.2 ∧ *p*-value < 0.05 (where |FC| is the absolute fold change of each gene) allowed us to estimate how significantly the diets are affecting the adipose tissue processes. It also showed how the expression intensities and processes differ between the two diets. Data was corrected for multiple testing using the q-value method [18].

### 2.5. Network Inference and Clustering

In order to study and visualise the gene expression over time, we opted to generate co-expression networks. To maintain correlation values of gene pairs across the three time points, we applied and modified the correlation method used in the Dynamically Co-expressed Neighbourhood (DCeN) algorithm [19]. The DCeN algorithm was developed for analysing time series gene expression data and calculates the correlations within individuals first which are then averaged per group. In our modification (see R implementation in Supplementary File, section Source Code), we calculate the signed correlations for generating the network for each diet based on a single input data group, using an absolute correlation value cut-off at 0.6 (|corr.| ≥ 0.6) in at least 25% of the samples in each group, leaving only strong correlations. This is performed for each diet, after which an overlap network is constructed by taking the intersection of the edges present in the respective correlation networks.

Frobenius norm was used to address the inter-individual gene expression and expression pattern variability within the respective diets. The Frobenius norm measures the square root of the sum of square differences between the correlation matrix of the diet and the correlation matrix of the individual. The difference presents a distance dimension to compare how “distant” an individual is compared to the whole group in the diet itself. Mathematically, for two correlation matrices A and B with *n* gene correlations, the Frobenius norm will be calculated as

(1) $$||A-{B||}_{F}=\sqrt{\sum_{i=1}^{n}\sum_{j=1}^{n}{\left|a_{ij}-b_{ij}\right|}^{2}}.$$

Topological clustering was done on the co-expression networks using the GLay community cluster algorithm [20] through the ClusterMaker app [21] in Cytoscape [16]. Since the networks were already based on correlated gene expression patterns over time, closely connected gene clusters in the network represented groups of genes having a highly similar or highly dissimilar expression pattern over time, and thus provided links between the processes in which the genes are involved. The log${}_{2}$ fold change of genes in the respective topological gene clusters of each diet was also plotted for each cluster to visually represent the clustering patterns over time, shown in Figures S2 and S3.

### 2.6. Gene Ontology and Pathway Enrichment

Gene Ontology (GO) enrichment was performed on both the complete networks as well as the network clusters using the ClueGO app [22] in Cytoscape. For the gene clusters, the settings in ClueGO were modified to allow any number or percentage of genes in any level of GO (0–20 in ClueGO) with the *p*-values of the pathways ≤0.05. For the complete network, the settings only differed in having at least 3 in number, or at least 4%, of genes in the respective GO terms. Pathway enrichment was performed using the over-representation analysis module at ConsensusPathDB [23], selecting all available pathway databases using a minimum gene overlap of 2 and a *p*-value ≤ 0.01.

### 2.7. Software and Libraries

ArrayAnalysis.org [14] was used for QC and normalisation, using custom chip definition file (CDF) annotation from BrainArray (version 19.0.0, ENSG). R [15] v3.2.3 was used with limma v3.26.5 package [24] for the differential gene expression analysis and qvalue v2.2.2 package [18] for the false discovery rate (FDR) analysis. Cytoscape [16] v3.4.0 was used for visualisation of the networks. Clustering was done using the ClusterMaker2 app [21] v0.9.5 in Cytoscape. ClueGO app [22] v2.3.2 in Cytoscape was used for GO enrichment, and ConsensusPathDB [25,26] version 31 was used for the pathway enrichment.

### 2.8. Data Availability

All relevant data is within the paper and its Supporting Information files. Gene expression data for the Yoyo study is accessible at Gene Expression Omnibus (accession number GSE77962).

## 3. Results

### 3.1. Data Normalisation and Filtration

The microarray gene expression data was obtained from a weight loss study comparing a low calorie diet (LCD) and a very low calorie diet (VLCD) [27]. In addition, 138 samples were used from the study—46 individuals across three time points. The three time points measured gene expression in the adipose tissue before weight loss, after weight loss, and after weight maintenance, respectively. Starting with the raw expression data of the 46 individuals, the QC analysis results showed two microarray samples as outliers (Figure S1). These outliers were two individuals from the VLCD diet group because their data at time point 1 (before weight loss) differed from the data cluster of the remaining VLCD members, whereas the data at the other two time points did not. This inconsistency led to the removal of the data from these two individuals across all three time points to remove any skewness in expression intensities or patterns introduced by the possibly erroneous data at these first time point samples. The normalised expression data including 21,641 genes was filtered for background gene expression leaving a total of 18,113 unique genes.

### 3.2. Differential Expression

**Figure 2:**
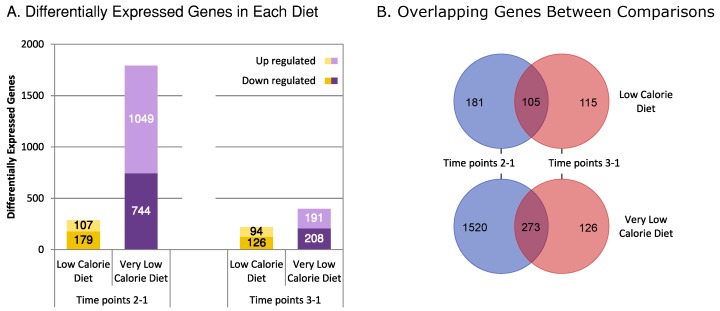
The number of differentially expressed genes in each diet. (**A**) The number of up and downregulated genes along the two time point comparisons. Time points 2-1: After Weight Loss—Before Weight Loss, and time points 3-1: After Weight Maintenance—Before Weight Loss; (**B**) the number of differentially expressed genes overlapping between the two comparisons within each diet.

The differential expression analysis was performed within individuals, between expressions after weight loss and before weight loss (time points 2-1); after weight maintenance and before weight loss (time points 3-1); and after weight maintenance and after weight loss (time points 3-2). The time points 3-2 analysis for LCD came up with very high local false discovery rate (FDR) for *p*-value < 0.05, implying a high chance of false positives even in significant results. Thus, this time point difference was not included in further analyses for either diet. In Figure 2, results of the differential gene expression analyses between the different time points in the different diets are shown and compared. A total of 286 genes were significantly differentially expressed between time points 1 and 2 for LCD, and 1793 genes for VLCD in the same period. These numbers goes down to 220 for LCD and 399 for VLCD respectively between time points 1 and 3. The difference in number of significant differentially expressed genes between LCD and VLCD during the weight loss period indicates that the pattern of expression in VLCD is more perturbed than in LCD in response to stronger caloric restriction. Furthermore, not all genes in the two comparisons within respective diets were the same, with some genes being significantly perturbed in either the weight loss period, or the weight maintenance period, but not in both.

### 3.3. Correlation and Overlap Networks

The differentially expressed genes of each diet were then used to create expression data subsets for each diet from the filtered dataset. These subsets were fed into the modified DCeN algorithm (complete procedure given in the Materials and Methods section) to generate the correlation matrices, using an absolute Pearson correlation of ≥0.6 as the cut-off. The generated co-expression network for the LCD diet contained 123 genes (nodes) and 250 correlations between these genes (edges). Forty-one of the 250 edges have negative correlation values. The positive correlations indicate highly similar expression patterns across the three time points, while the negative correlations indicate mirrored expression patterns. The LCD correlation network is shown in Figure S2. For the VLCD diet, the network contained 1382 nodes and 35,791 edges, of which 11,270 edges showed negative correlations between their respective genes. The VLCD correlation network is shown in Figure S3.

**Figure 3:**
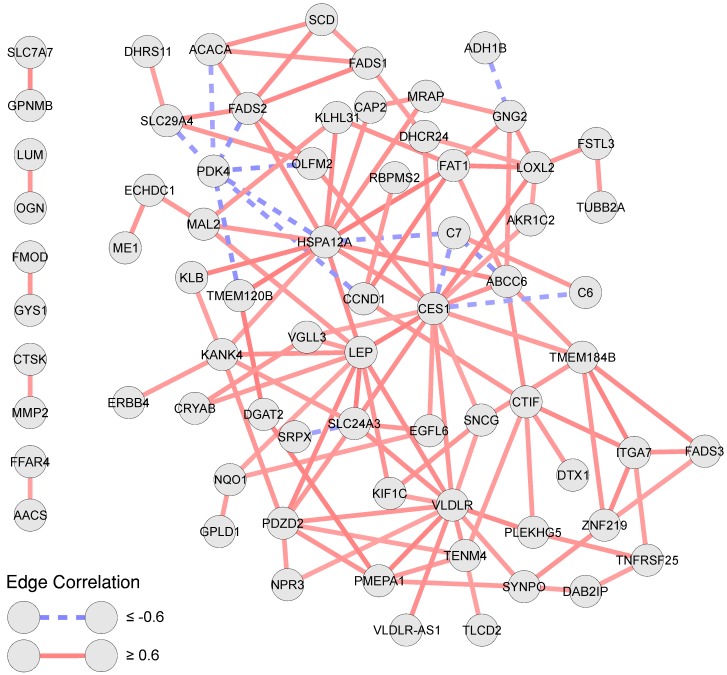
Overlap network showing the intersection of the edges of the low calorie diet (LCD) and the very low calories diet (VLCD) correlation networks. The intersection only depends on the sign/direction of the correlation (positive or negative), and not the exact value of the correlation.

To find the common mechanisms, irrespective of the intensity and duration of caloric restriction, we generated an overlap network of the two diets. The overlap network is constructed by finding the gene pairs (edges) that are common between the two networks and correlated in the same direction (positive or negative). The overlap network shown in Figure 3 consisted of a single large connected component and five separate pairs correlated genes. The complete network contained 71 genes and 127 correlations between those genes, of which only 13 correlations were negative. The flexibility of the workflow to use the high level of sample variability was checked by comparing the correlation matrices of each individual with the correlation matrices of their respective diet group using the Frobenius norm of the distance matrix. The plot of the calculated Frobenius distances showing each individual in the respective diet is provided as Figure S4. The plot includes certain individuals having a higher distance from the respective diets, which shows that these individuals, despite the variability in expression data, were still part of the construction of the correlation network of the respective diet.

### 3.4. Enrichment and Clustering Results

**Figure 4:**
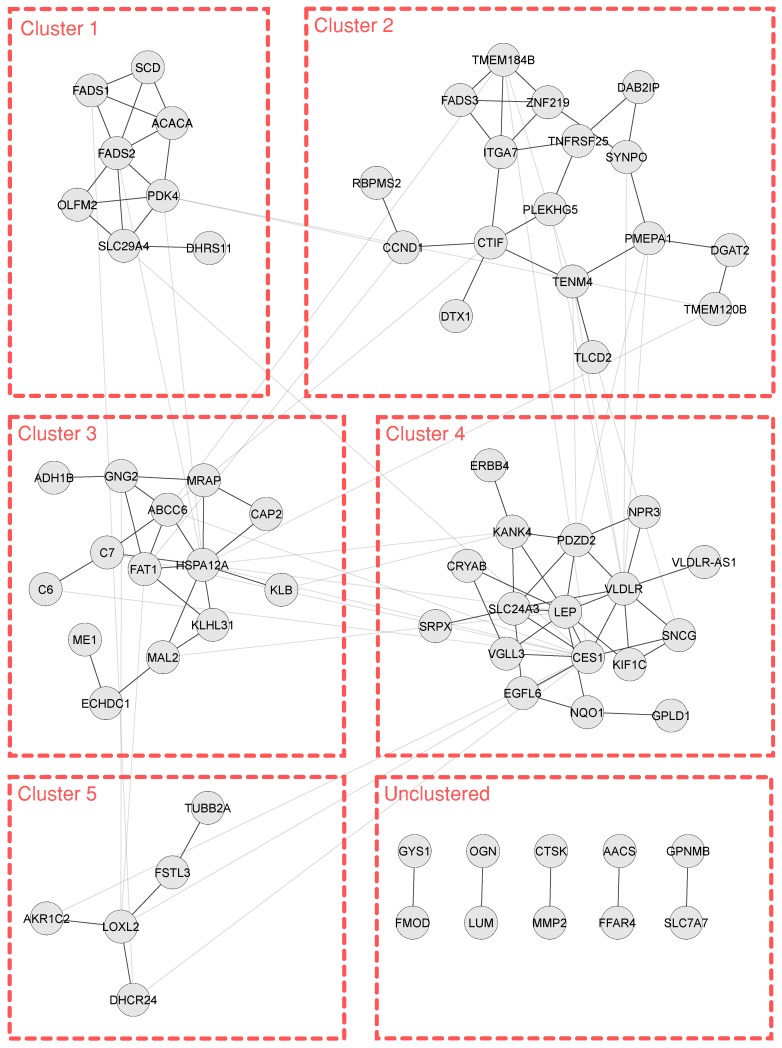
GLay community clusters of the overlap network. Faded edges show the edges removed by the algorithm to generate the “community” of genes based on the topology.

**Figure 5:**
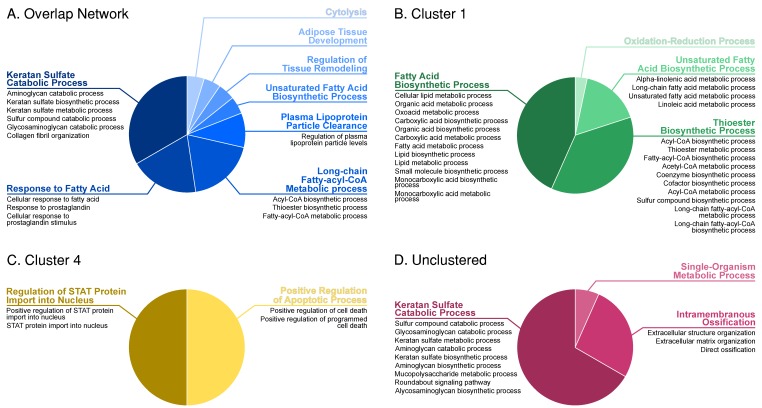
Gene ontology term pie charts constructed using the ClueGO results. Each pie chart contains major GO terms as slices, with sub-terms listed underneath each. (**A**) GO term pie chart for the overlap network; (**B**) GO term pie chart for Cluster 1; (**C**) GO term pie chart for Cluster 4; (**D**) GO term pie chart for un-clustered gene pairs.

The overlap network was then used for Gene Ontology (GO) enrichment analysis. The most significant classes ranged from metabolic processes such as long chain fatty-acyl-CoA metabolic process (GOID: 35336) and keratan sulphate catabolic process (GOID: 42340), to non-metabolic processes such as adipose tissue development (GOID: 60612) and regulation of tissue remodelling (GOID: 34103). Using the topological clustering method described in the methods section, the overlap network was clustered into five clusters, and a set of five un-clustered paired genes shown in Figure 4. Cluster 1 consists of eight genes, followed by 17 in Cluster 2, 14 in Cluster 3, 17 in Cluster 4, and five in Cluster 5, with the five unconnected paired correlations remaining unclustered. Clusters 1 and 4 provided additional significant enrichment terms, such as thioester biosynthetic process (GOID: 35384) and regulation of signal transducer and activator of transcription *STAT* protein input into nucleus (GOID: 2000364) respectively, adding a new dimension of process regulation information to the network. The five un-clustered gene pairs were enriched together and predominantly targeted the keratan sulphate catabolic process (GOID: 42340). Clusters 2 and 3 did not provide any significant enriched terms. Figure 5 shows these resultant GO terms as pie charts. Of the 71 genes in the overlap network, carboxylesterase 1 (*CES1*), heat shock protein family A member 12A (*HSPA12A*), very low density lipoprotein receptor (*VLDLR*) and leptin (*LEP*) showed a high degree centrality of 15, 14, 12 and 11, respectively. Expectedly, these high degree genes are parts of clusters, with *CES1*, *VLDLR*, and *LEP* belonging to Cluster 4, while *HSPA12A* acts as the hub node in Cluster 3.

Additionally, the expression patterns of the genes were plotted for each cluster using their log${}_{2}$ fold change within each diet. The plots, provided as Figure S5, show either the similarity or the mirroring of the expression pattern of each gene with respect to the rest of the genes within the cluster. Interestingly, the plots show that much of the expression pattern clustering is dependent on the change in expression pattern between the first two time points (during weight loss) with variations seen in the patterns between the last two time points (during weight maintenance). Across the 71 genes of the overlap network, *PDK4*, *C6*, *C7*, *ADH1B*, *SRPX*, *GPNMB*, *SLC7A7*, *MMP2*, *OGN*, *LUM*, and *CTSK* were found to be upregulated after weight loss, with the remaining genes downregulated. After weight maintenance, however, the gene expression pattern has variations with many gene expression patterns showing a reversal in trend but some genes also maintaining their expression patterns achieved during weight loss such as *MMP2*, *OGN* and *LUM*.

## 4. Discussion

In this article, we presented a network biology workflow to find genes of interest using gene expression data for targeted analysis of processes and pathways affected during weight loss. Our data-driven workflow is applicable to any time series expression data. One of the reasons for the construction of the workflow was the previously reported poor performance of existing correlation calculation methods on human time series expression data [19]. Essentially, most standard techniques generate correlations between the average expression of the genes across the whole group. This methodology works perfectly for homogeneous datasets/groups, but, for heterogeneous groups, this methodology fails as the average gene expression of the group is dampened because of the high variability in the measurements. The dynamically co-expressed neighbourhoods (DCeN) algorithm created by Elo and Schwikowski was specifically made to address this issue by calculating gene–gene correlations within individual members of the dataset, and then averaging the correlations, thus preserving the variability in the data while also generating meaningful correlations [19]. In our article, we modified the DCeN algorithm to focus on a single expression group at a time to generate the correlation matrices of the diets individually.

Based on the high number of differentially expressed genes in VLCD, the resultant network for VLCD was also much larger in terms of the number of genes (nodes) and correlated expression patterns (edges) than the LCD network. The difference in size of the networks, coupled with the number of differentially expressed genes, clearly shows that the adipose tissue gene expression is affected in both diets and that VLCD causes a larger perturbation of the adipose tissue. The overlap network, constructed via the edge intersection of the co-expression networks of the diets, provides a set of genes having the same expression pattern in both diets. This set of genes and their expression pattern represents a behaviour, which, based on our results, is likely to always occur with weight loss irrespective of the caloric restriction, or at least for diets between 1250 kcal/day and 500 kcal/day. In that respect, this set is ideal for construction of adipose tissue regulatory behaviours involved in weight loss, and can be then extended to other differentially expressed genes found in the respective diets, as can be seen by the processes found and highlighted in the results of the GO enrichment analysis.

In addition, we also explored pathway enrichment methods which gave functionally very similar results to GO enrichment. The complete list of enriched pathways is provided as Table S4. Most of the enriched pathways and processes relate to metabolism and have been found crucial in obesity, T2DM, CVD and MetS [4,28]. The enriched results include fatty acid biosynthesis pathways such as the downregulation of the acetyl-CoA metabolic network, previously described in [29]. We also confirmed the involvement of signalling pathways such as *AMPK* [30,31] and *PPAR* signalling [32,33]. Omega-3 and Omega-6 fatty acid metabolism pathways, as well as the complement system/cascade, were also found to be enriched in our results. Omega-3 have been proposed to be involved in lipid metabolism and adipokine regulation, both affected themselves in obesity and metabolic syndromes [34]. Omega-6 fatty acids have been found to be involved in anti-inflammatory effects [35]. However, high Omega-6 to Omega-3 ratio has also been found to increase the risk for obesity [36]. The complement cascade/system has been found to be dysregulated in obesity and associated co-morbidities, linking inflammatory effects, insulin resistance and impaired metabolism [37]. Additionally, other pathways such as ‘reversal of insulin resistance by leptin’ were also enriched for the overlap network, indicating that certain beneficial pathways are triggered irrespective of the level and duration of caloric restriction. However, the overlap network only indicates a core set of pathways, and the intensity of the effect of the respective pathways and biological processes will differ when taken together with the extended networks unique to each diet.

Detailed literature study of the 71 genes in the overlap network yielded additional details regarding the involvement of these genes and their products in adipose tissue metabolism. Interestingly, the clusters of these genes showed unifying themes for most of the members of the respective clusters, indicating possible points of cross-talk of different cellular processes in the larger backdrop of adipose tissue metabolism and energy homeostasis. Additionally, some genes and their products have not been profiled for any particular cellular task or process within the context of obesity or adipose tissue metabolism. However, since these genes were found to be part of their respective clusters, the possible cross-talks of these genes allows for new avenues of research. The gene symbols of all the clustered and unclustered genes of the overlap network are provided as Table S5. In Cluster 1, the pyruvate dehydrogenase kinase 4 (*PDK4*) and acetyl-coenzyme A carboxylase 1 (*ACACA*) genes are clustered together, representing the upstream regulators of the tricarboxylic acid (TCA) cycle. The protein kinase *PDK4* has previously been identified as an inhibitory regulator of pyruvate dehydrogenase complex (*PDC*) [38] which converts pyruvate and co-enzyme A (CoA) into acetyl-CoA for the TCA cycle. On the other hand, *ACACA* uses acetyl-CoA for fatty acid biosynthesis by converting it to malonyl-CoA [39]. Stearoyl-CoA desaturase (*SCD*), fatty acid desaturase 1 and 2 (*FADS1* and *FADS2* respectively), were also clustered with *PDK4* and *ACACA*. The protein *SCD* has been shown to take part in triglyceride storage in white adipose tissue [40]. Furthermore, *SCD*, *FADS1* and *FADS2* are involved in the desaturation of fatty acids, and, as such, are linked to de novo fatty acid synthesis.

The genes constituting the second cluster of the overlap network (Cluster 2 in Figure 4) are also predominantly linked with metabolism, lipogenesis and lipolysis, in particular diacylglycerol O-acyltransferase 2 (*DGAT2*), zinc finger protein 219 (*ZNF219*), and fatty acid desaturase 3 (*FADS3*). However, considering the filtering of the genes and the study design of the source data, which is to be expected. Next to genes related to this common theme, links to tissue proliferation and differentiation were found in the cluster as well. G1/S-specific cyclin-D1 (*CCND1*), integrin alpha-7 precursor (*ITGA7*), transmembrane protein 120B (*TMEM120B*) and transmembrane protein 184B (*TMEM184B*) all have been associated with tissue proliferation and differentiation [41,42].

The genes in Cluster 3 represent closer links to inter-cell signalling and immune system response apart from adipose tissue metabolism. Complement component *C6* and *C7* play a role in innate immune response by forming the terminal membrane attack complex (MAC) [43,44]. FAT atypical cadherin 1 (*FAT*) is involved in cell–cell signalling and has been shown to be affected by diet [45]. Adenylate cyclase-associated protein 2 (*CAP2*), kelch-like family member 31 (*KLHL31*) and klotho beta (*KLB*) are also involved in signal transduction through hedgehog, *IFN-JAK-STAT* and *MAPK* pathways [46,47]. Except for these and alcohol dehydrogenase 1B (*ADH1B*), malic enzyme 1 (*ME1*) and ethylmalonyl-CoA decarboxylase (*ECHDC1*), other expressed genes in the cluster have little known information in the context of obesity and chronic diseases.

Relative to the previous three clusters, the genes in Cluster 4 show a more diverse profile in terms of intra-, inter- and extra-cellular biological processes. Perhaps the most important gene expression in the cluster is that of leptin (*LEP*), an adipokine responsible for organism-wide signalling through multiple signalling pathways [48,49]. Through these signalling pathways, *LEP* has been implicated in regulation of tissue and organism-wide metabolism, energy homeostasis, fat storage and inflammation [50,51]. The organism-wide effects are extended by a very low density lipoprotein receptor (*VLDLR*), which facilitates removal of circulating very low density lipoproteins [52], and has also been found to induce adipose tissue inflammation [53]. Other genes found in this cluster that are known to be involved in signal transduction include synuclein gamma (*SNCG*) [54], and erb-B2 receptor tyrosine kinase 4 (*ERBB4*) [55,56].

Cluster 5 was the smallest of all clusters, consisting of only five members: follistatin like 3 (*FSTL3*), 24-dehydrocholesterol reductase (*DHCR24*), lysyl oxidase like 2 (*LOXL2*), tubulin beta 2A class IIa (*TUBB2A*) and aldo-keto reductase family 1 member C2 (*AKR1C2*). *FSTL3* is a high-affinity inhibitor of transforming growth factor beta (TGFβ) family members, including activin A and myostatin, and thus is involved in modulating glucose homeostasis [57]. *DHCR24* binds to and protects *p53* from degradation, shielding the cell from oxidative stress [58]. *LOXL2* and *TUBB2A* appear to be involved with cellular and tissue morphology via extracellular matrix cross-linking [59] and microtubule component synthesis, respectively. *AKR1C2* is known to be associated with central obesity and association with long-term weight gain in men has been suggested [60,61]. In addition, changes of the *AKR1C2* protein in the adipose tissue during weight maintenance were found to positively correlate with body weight, waist, BMI and plasma low density lipoprotein (LDL), and to correlate negatively with plasma *LEP* [62]. Nevertheless, the exact role of the expression of this gene in terms of cellular processes related to obesity is unknown [60].

In summary, our study found several pathways known to be involved with obesity and associated co-morbidities, along with clusters of genes representing different but related molecular and biochemical processes in the adipose tissue cells. Many of these pathways have been known to be associated with several dysregulations in obesity; however, the interactions and cross-talk between these pathways and cellular functions need to be further explored to better understand the cellular and tissue wide dysregulation in obesity and associated chronic diseases. Collectively, the presented results provide directions for future research and exploration of obesity related chronic diseases through predictive modelling and analyses.

## 5. Conclusions

In conclusion, our article presents a workflow for finding candidate regulatory genes and processes using differential gene expression and expression pattern over time using a time series dataset. We have shown that this workflow is able to isolate several biological processes and pathways having known links with obesity, T2DM, CVD, and metabolic syndrome, allowing future analyses and predictive modelling focused on these particular biological processes. The workflow presented here is flexible by design and uses only freely available tools that can be easily connected. It is applicable to any form of time series expression data such as RNA-seq data and non-coding RNA expression arrays and it effectively accommodates inter-sample variability when constructing the correlation networks. This allows network based analyses of human intervention studies and datasets for which this was previously difficult.

## Supplementary Materials

The following are available online at http://www.mdpi.com/2073-4425/9/11/525/s1, Figure S1: Principal component analysis of the raw gene expression data, Figure S2: Correlation network generated for the low calorie diet (LCD), Figure S3: Correlation network generated for the very low calorie diet (VLCD), Figure S4: Frobenius norm plot, Figure S5: Gene expression patterns of the respective genes in each topological cluster of the overlap network, Table S1: Results from the robustness analysis, Table S2: Results from the leave-n-out cross-validation analysis, Table S3: Results from the ConsensusPathDB analysis, Table S4: List of genes in the respective clusters from the overlap network.
