# Supplementary material for: Profiling Cellular Processes in Adipose Tissue during Weight Loss Using Time Series Gene Expression
Source: Genes (Basel). 2018 Oct 29;9(11):525. doi: 10.3390/genes9110525 (PMC6266822; doi:10.3390/genes9110525)
Supplement: Supplementary file 1 [file genes-09-00525-s001.pdf]

# Profiling cellular processes in adipose tissue during weight loss using time series gene expression

**Samar H.K. Tareen[1,*], Michiel E. Adriaens[1], Ilja C.W. Arts[1,2], Theo M. de Kok[1,3], Roel G. Vink[4], Nadia J.T. Roumans[4], Marleen A. van Baak[4], Edwin C.M. Mariman[4], Chris T. Evelo[1,5], and Martina Kutmon[1,5]**

[1]Maastricht Centre for Systems Biology (MaCSBio), Maastricht University, Maastricht, the Netherlands

[2]Department of Epidemiology, CARIM School for Cardiovascular Diseases, Maastricht University, Maastricht, the Netherlands

[3]Department of Toxicogenomics, GROW School of Oncology and Developmental Biology, Maastricht University, Maastricht, the Netherlands

[4]Department of Human Biology, NUTRIM Research School, Maastricht University, Maastricht, the Netherlands

[5]Department of Bioinformatics - BiGCaT, NUTRIM Research School, Maastricht University, Maastricht, the Netherlands

*samar.tareen@maastrichtuniversity.nl

# Supplementary Information

**Figures** 2

Figure S1: Principal component analysis of the raw gene expression data of the 46 individuals, showing the outlying samples highlighted in red circles. 2

Figure S2: Correlation network generated for the low calorie diet (LCD). 3

Figure S3: Correlation network generated for the very low calorie diet (VLCD). 3

Figure S4: Frobenius norm plot showing the norm distance of the correlation network of the 44 individuals with the respective diet-wide correlation network. 4

Figure S5: Gene expression patterns of the respective genes in each topological cluster of the overlap network, based on the fold change in the respective diets. Also shows the five un-clustered paired correlated genes. 5

**Source Code** 5

**Tables** 7

Table S1: Results from the robustness analysis using the minimum %age individuals having the same correlation direction (for |corr.| ≥ 0.6). 7

Table S2: Results from the leave-n-out cross-validation analysis for n= 1 and 2 (max 10% of total samples). The correlation criteria is |corr.| ≥ 0.6 in at least 25% of the individuals having the same direction of the correlation. 8

Table S3: Results from the ConsensusPathDB analysis on the overlap network. BioCarta leptin pathway discussed in the discussion section of the article is highlighted in red. 8

Table S4: List of genes in the respective clusters from the overlap network, represented here in HGNC symbols. 12

# Figures

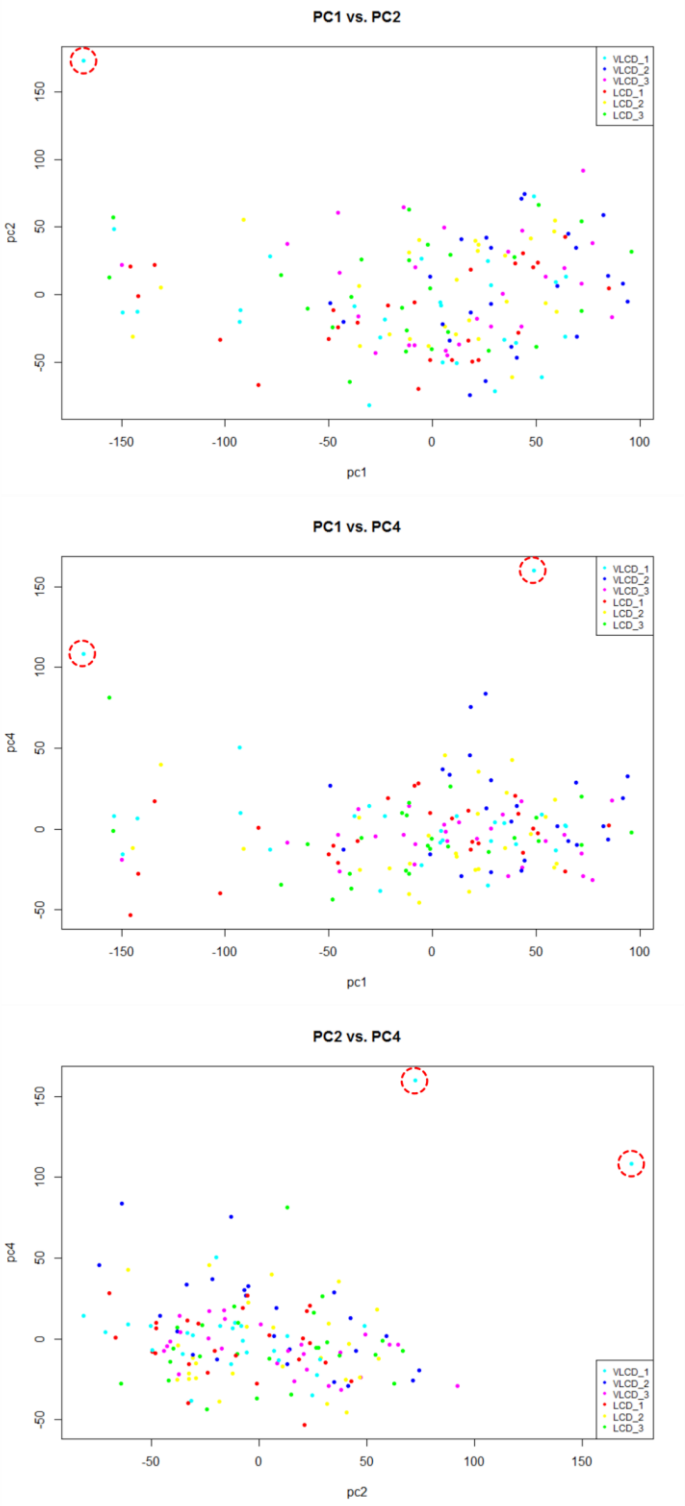


Figure S1: Principal component analysis of the raw gene expression data of the 46 individuals, showing the outlying samples highlighted in red circles.

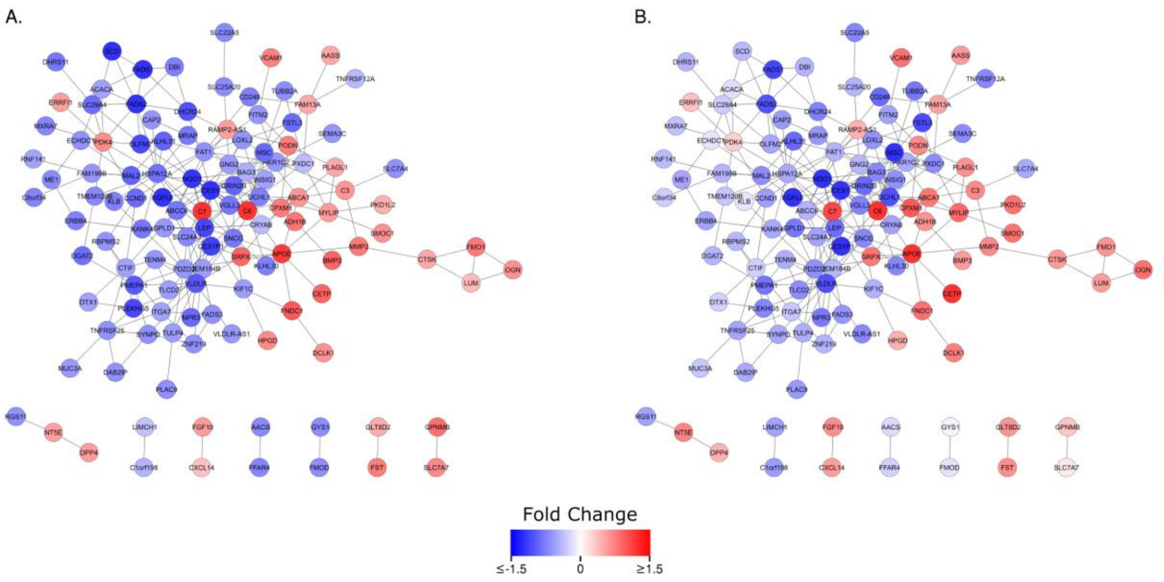


Figure S2: Correlation network generated for the low calorie diet (LCD).

A) Fold change for time points 2-1
B) Fold change for time points 3-1

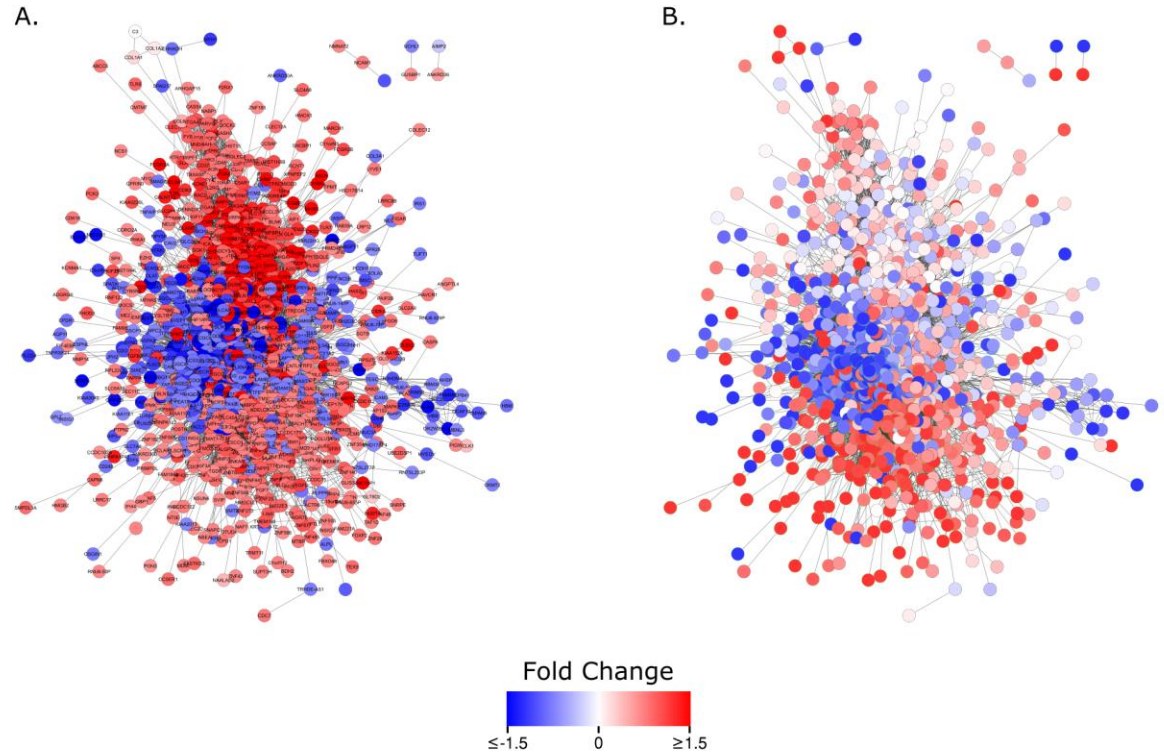


Figure S3: Correlation network generated for the very low calorie diet (VLCD).

A) Fold change for time points 2-1
B) Fold change for time points 3-1

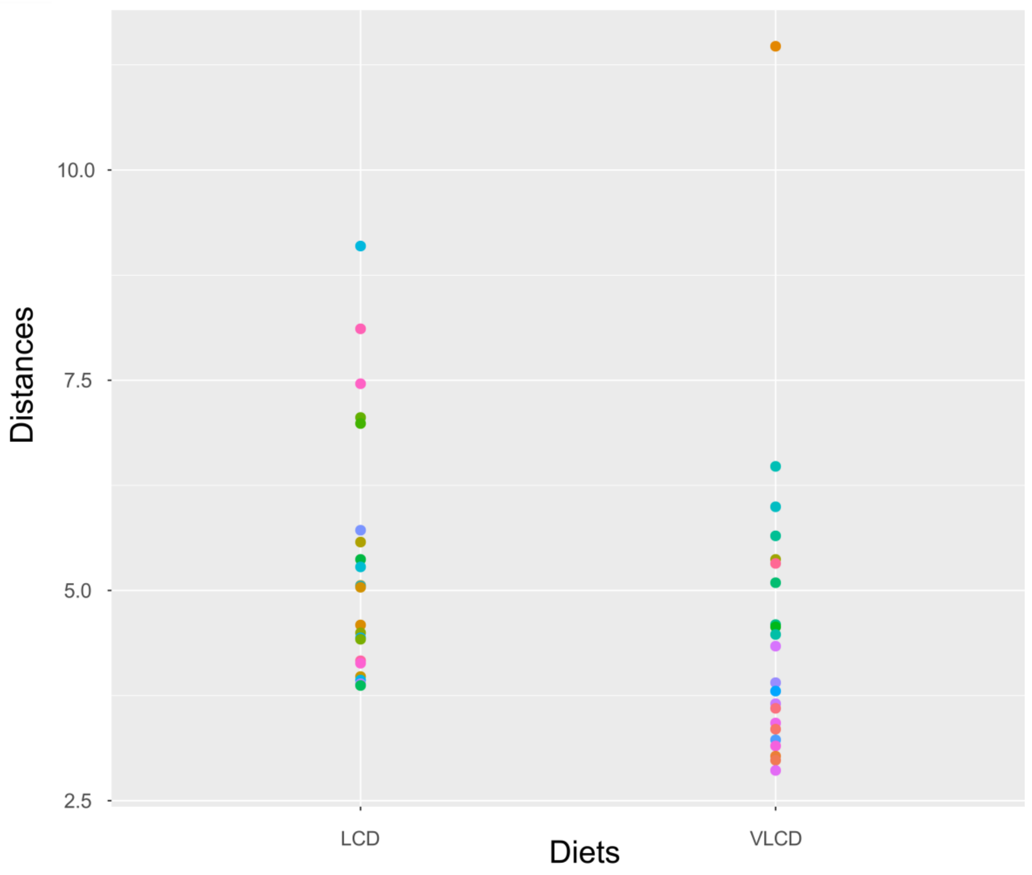


Figure S4: Frobenius norm plot showing the norm distance of the correlation network of the 44 individuals with the respective diet-wide correlation network.

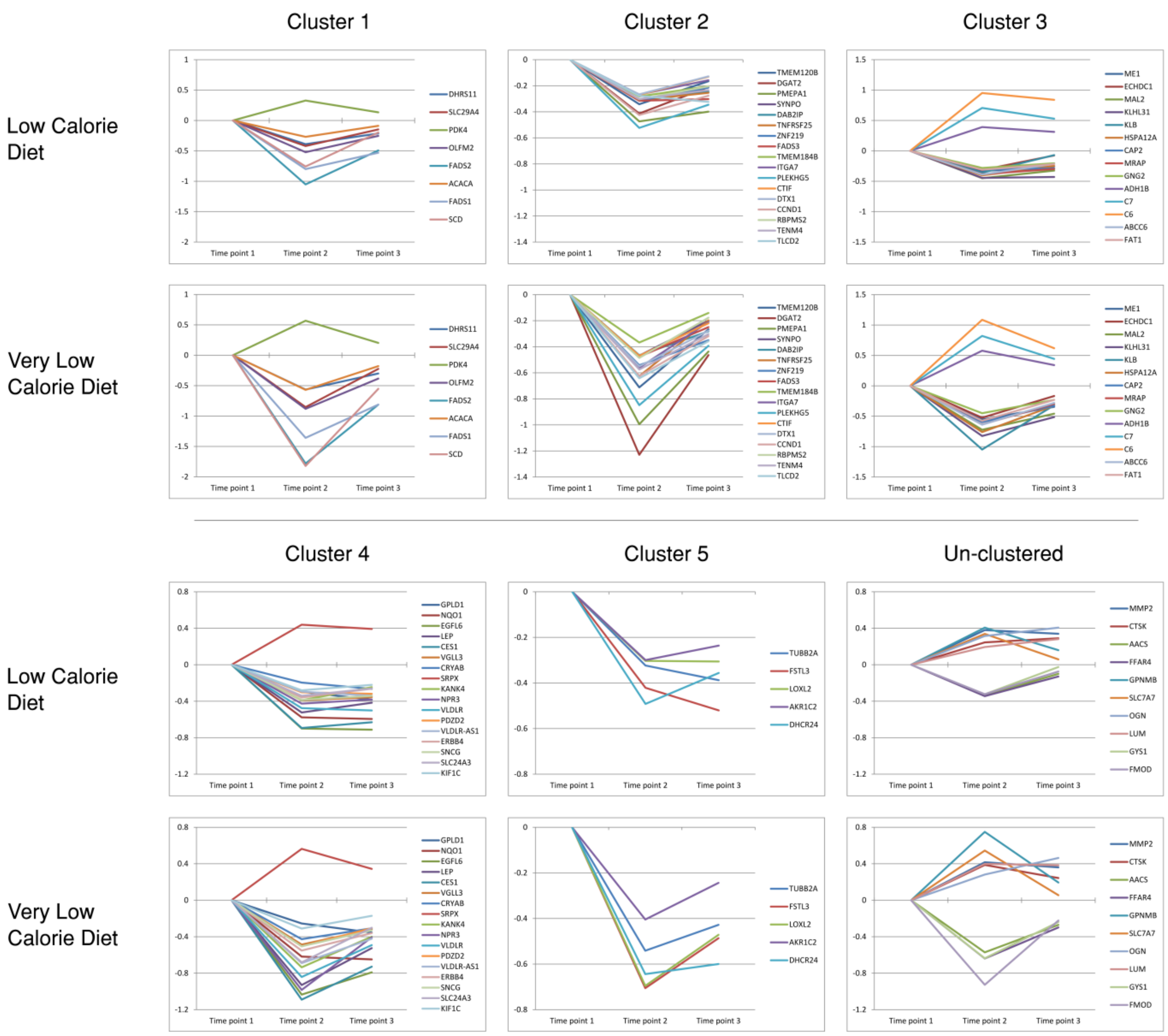


Figure S5: Gene expression patterns of the respective genes in each topological cluster of the overlap network, based on the fold change in the respective diets. Also shows the five un-clustered paired correlated genes.

# Source Code

```
#####
###
#Modified DCeN algorithm for the creation of correlation networks.
#
#Credit for creating the original algorithm goes to:
#        Elo LL, Schwikowski B (2013) Analysis of Time-Resolved Gene Expression 
Measurements across Individuals. PLoS ONE 8(12): e82340. 
doi:10.1371/journal.pone.0082340
#
#Modified by Samar Tareen for use with single group/stimulus data over multiple 
individuals
#Submitted with Manuscript ID: as Appendix material
#
#For complete details of each section of the algorithm, we refer to the original 
publication
#
```

```
###


#Function name 'tsCorr' (time series correlation); Takes
#       1. dataframe as input data
#       2. filter.corr=0.7 as default minimum correlation cutoff for 1-filter.corr in
positive and -(1-filter.corr) in negative correlations
#       3. filter.n=0.25 as minimum percentage of individuals in which the
correlations should hold

tsCorr <- function(data, filter.corr=0.7, filter.n=0.25) {

    ## Count significant correlations and determine prefiltering for positive
correlations
    posFilt<- matrix(nrow=nrow(data[[1]]), ncol=nrow(data[[1]]))
    posFilt[]<- 0
    negFilt<- matrix(nrow=nrow(data[[1]]), ncol=nrow(data[[1]]))
    negFilt[]<- 0
    avgCorr<- matrix(nrow=nrow(data[[1]]), ncol=nrow(data[[1]]))
    avgCorr[]<- 0

    for(i in 1:length(data)) {
        C1<- cor(t(data[[i]]))
        C1<- C1 * sqrt(ncol(data[[i]])-2) / sqrt(1 - C1*C1)
        C1<- (1 - 2 * pt(-abs(C1), ncol(data[[i]])-2, lower.tail = TRUE, log.p =
FALSE))*sign(C1)

        avgCorr<- avgCorr + C1

        posTemp <- C1
        posTemp[posTemp >  1-filter.corr]<- 1
        posTemp[posTemp <= 1-filter.corr]<- 0
        diag(posTemp)<- 0
        posFilt<- posFilt + posTemp
        rm(posTemp)

        negTemp <- C1
        negTemp[negTemp <  -(1-filter.corr)]<- -1
        negTemp[negTemp >= -(1-filter.corr)]<- 0
        diag(negTemp)<- 0
        negFilt<- negFilt + negTemp
        rm(negTemp)
    }
    rm(C1)
    gc()

    cutoff1<- ceiling(filter.n*length(data))
    posFilt[posFilt<cutoff1]<- 0
    A.pos<- pmax(posFilt)
    A.pos[A.pos>0]<- 1
    rm(posFilt)
    gc()

    negFilt<- abs(negFilt)
    cutoff1<- ceiling(filter.n*length(data))
    negFilt[negFilt<cutoff1]<- 0
    A.neg<- pmax(negFilt)
    A.neg[A.neg>0]<- 1
    rm(negFilt)
    gc()

    avgCorr<- avgCorr/length(data)
```

```
    C1<- avgCorr
    rm(avgCorr)
    gc()

    A<- pmax(A.pos,A.neg)
    C1[A==0]<- 0
    a<- rowSums(A)
    rm(A.pos)
    rm(A.neg)
    rm(A)
    gc()

    #in case the correlation network needs to be generated as output (tab delimited
text file)
    #write.table(C1, paste(getwd(), "/tsCorr.txt", sep=""), sep="\t", row.names = T,
col.names = T, quote=F)
    return(C1)
}

#####
```

# Tables

Table S1: Results from the robustness analysis using the minimum %age individuals having the same correlation direction (for |corr.| ≥ 0.6).

| %age minimum individuals | | LCD | VLCD |
|---|---|---|---|
| 25 | Total edges | 127 | 127 |
| | %age | 100.00 | 100.00 |
| 30 | Conserved edges | 127 | 126 |
| | %age | 100.00 | 99.21 |
| 35 | Conserved edges | 127 | 126 |
| | %age | 100.00 | 99.21 |
| 40 | Conserved edges | 125 | 126 |
| | %age | 98.43 | 99.21 |
| 45 | Conserved edges | 119 | 125 |
| | %age | 93.70 | 98.43 |
| 50 | Conserved edges | 108 | 120 |
| | %age | 85.04 | 94.49 |

Table S2: Results from the leave-n-out cross-validation analysis for n= 1 and 2 (max 10% of total samples) in the overlap network. The analysis here represents loss of sensitivity. The correlation criteria is |corr.| ≥ 0.6 in at least 25% of the individuals having the same direction of the correlation.

| **Leave-n-out, n=** | **# of combinations in each diet** | **Criteria: \|corr.\| ≥ 0.6 and min. 25% ind.s** | **LCD** | **VLCD** |
|---|---|---|---|---|
| 0 | 1 | Total edges | 127 | 127 |
| | | %age | 100.00 | 100.00 |
| 1 | 22 | Conserved edges | 85 | 117 |
| | | %age | 66.93 | 92.13 |
| 2 | 231 | Conserved edges | 52 | 104 |
| | | %age | 40.94 | 81.89 |

Table S3: Results from the leave-n-out cross-validation analysis for n= 1 and 2 (max 10% of total samples) in the correlation networks of the respective diets. The analysis here represents loss of specificity. The correlation criteria is |corr.| ≥ 0.6 in at least 25% of the individuals having the same direction of the correlation.

| **Leave-n-out, n=** | **# of combinations in each diet** | **Criteria: \|corr.\| ≥ 0.6 and min. 25% ind.s** | **LCD** | **VLCD** |
|---|---|---|---|---|
| 0 | 1 | Total edges | 149 | 1125 |
| 1 | 22 | New edges (avg. of all combinations) | 20 | 54 |
| 2 | 231 | New edges (avg. of all combinations) | ~36 | ~82 |

Table S4: Results from the ConsensusPathDB analysis on the overlap network. BioCarta leptin pathway discussed in the discussion section of the article is highlighted in red.

| **pathway** | **p-value** | **q-value** | **source** | **members_input_overlap** | **members_input_overlap_geneids** | **size** | **effective_size** |
|---|---|---|---|---|---|---|---|
| Omega-3 fatty acid metabolism | 4.84E-05 | 0.00781987 | EHMN | ENSG00000221968; ENSG00000134824; ENSG00000149485 | 3995; 3992; 9415 | 16 | 16 |
| Fatty Acid Biosynthesis | 0.000130585 | 0.00781987 | Wikipathways | ENSG00000099194; ENSG00000093144; ENSG00000278540 | 55862; 31; 6319 | 22 | 22 |
| Biosynthesis of unsaturated fatty acids - Homo sapiens | 0.000149689 | 0.00781987 | KEGG | ENSG00000099194; ENSG00000134824; | 3992; 6319; 9415 | 23 | 23 |

| | | | | | | | |
|---|---|---|---|---|---|---|---|
| (human) | | | | ENSG00000149485 | | | |
| AMPK signaling pathway - Homo sapiens (human) | 0.000235714 | 0.00781987 | KEGG | ENSG00000104812; ENSG00000099194; ENSG00000174697; ENSG00000110092; ENSG00000278540 | 6319; 31; 595; 3952; 2997 | 124 | 123 |
| Omega-6 fatty acid metabolism | 0.000272466 | 0.00781987 | EHMN | ENSG00000221968; ENSG00000134824; ENSG00000149485 | 3992; 3995; 9415 | 28 | 28 |
| Keratan sulfate biosynthesis | 0.000272466 | 0.00781987 | Reactome | ENSG00000139329; ENSG00000106809; ENSG00000122176 | 4060; 4969; 2331 | 28 | 28 |
| Alpha Linolenic Acid and Linoleic Acid Metabolism | 0.000304106 | 0.00781987 | SMPDB | ENSG00000134824; ENSG00000149485 | 9415; 3992 | 6 | 6 |
| Terminal pathway of complement | 0.000424502 | 0.008930523 | Reactome | ENSG00000112936; ENSG00000039537 | 730; 729 | 7 | 7 |
| Keratan sulfate/keratin metabolism | 0.000446526 | 0.008930523 | Reactome | ENSG00000139329; ENSG00000106809; ENSG00000122176 | 4060; 4969; 2331 | 33 | 33 |
| Nuclear Receptors Meta-Pathway | 0.000514786 | 0.009234772 | Wikipathways | ENSG00000065833; ENSG00000099194; ENSG00000198848; ENSG00000110092; ENSG00000004799; ENSG00000112186; ENSG00000181019 | 4199; 595; 1066; 6319; 1728; 10486; 5166 | 314 | 313 |
| Linoleic acid (LA) metabolism | 0.000564347 | 0.009234772 | Reactome | ENSG00000134824; ENSG00000149485 | 3992; 9415 | 8 | 8 |
| reversal of insulin resistance by leptin | 0.000723467 | 0.010852004 | BioCarta | ENSG00000174697; ENSG00000278540 | 3952; 31 | 9 | 9 |
| alternative | 0.000901689 | 0.012484929 | BioCarta | ENSG000001 | 730; 729 | 10 | 10 |

| | | | | | | | |
|---|---|---|---|---|---|---|---|
| complement pathway | | | | 12936; ENSG00000039537 | | | |
| Extracellular matrix organization | 0.001202898 | 0.015465837 | Reactome | ENSG00000135424; ENSG00000143387; ENSG00000134013; ENSG00000139329; ENSG00000087245; ENSG00000122176 | 4017; 3679; 2331; 4060; 4313; 1513 | 264 | 263 |
| lectin induced complement pathway | 0.001314758 | 0.01549266 | BioCarta | ENSG00000112936; ENSG00000039537 | 730; 729 | 12 | 12 |
| alpha-linolenic acid (ALA) metabolism | 0.001549266 | 0.01549266 | Reactome | ENSG00000134824; ENSG00000149485 | 3992; 9415 | 13 | 13 |
| alpha-linolenic (omega3) and linoleic (omega6) acid metabolism | 0.001549266 | 0.01549266 | Reactome | ENSG00000134824; ENSG00000149485 | 3992; 9415 | 13 | 13 |
| icosapentaenoate biosynthesis II (metazoa) | 0.001549266 | 0.01549266 | HumanCyc | ENSG00000134824; ENSG00000149485 | 3992; 9415 | 13 | 13 |
| classical complement pathway | 0.001802198 | 0.017073456 | BioCarta | ENSG00000112936; ENSG00000039537 | 730; 729 | 14 | 14 |
| ECM proteoglycans | 0.002000476 | 0.018004287 | Reactome | ENSG00000139329; ENSG00000135424; ENSG00000122176 | 4060; 3679; 2331 | 56 | 55 |
| Complement Activation, Classical Pathway | 0.00266988 | 0.022884683 | Wikipathways | ENSG00000112936; ENSG00000039537 | 730; 729 | 17 | 17 |
| AMPK Signaling | 0.003665435 | 0.026814921 | Wikipathways | ENSG00000104812; ENSG00000174697; ENSG00000278540 | 3952; 31; 2997 | 68 | 68 |
| PPAR signaling pathway - Homo sapiens (human) | 0.003819792 | 0.026814921 | KEGG | ENSG00000065833; ENSG00000099194; ENSG00000134824 | 6319; 9415; 4199 | 69 | 69 |
| Linoleate metabolism | 0.004306497 | 0.026814921 | EHMN | ENSG00000221968; | 9415; 3992; 3995 | 72 | 72 |

| | | | | | | | |
|---|---|---|---|---|---|---|---|
| | | | | ENSG00000134824; ENSG00000149485 | | | |
| Pyruvate Dehydrogenase Complex Deficiency | 0.004469153 | 0.026814921 | SMPDB | ENSG00000065833; ENSG00000278540 | 4199; 31 | 22 | 22 |
| Primary hyperoxaluria II, PH2 | 0.004469153 | 0.026814921 | SMPDB | ENSG00000065833; ENSG00000278540 | 4199; 31 | 22 | 22 |
| Pyruvate kinase deficiency | 0.004469153 | 0.026814921 | SMPDB | ENSG00000065833; ENSG00000278540 | 4199; 31 | 22 | 22 |
| Leigh Syndrome | 0.004469153 | 0.026814921 | SMPDB | ENSG00000065833; ENSG00000278540 | 4199; 31 | 22 | 22 |
| Pyruvate Metabolism | 0.004469153 | 0.026814921 | SMPDB | ENSG00000065833; ENSG00000278540 | 4199; 31 | 22 | 22 |
| Pyruvate Decarboxylase E1 Component Deficiency (PDHE1 Deficiency) | 0.004469153 | 0.026814921 | SMPDB | ENSG00000065833; ENSG00000278540 | 4199; 31 | 22 | 22 |
| Leptin signaling pathway | 0.005011674 | 0.028826678 | Wikipathways | ENSG00000174697; ENSG00000110092; ENSG00000278540 | 595; 3952; 31 | 76 | 76 |
| Metabolism | 0.005124743 | 0.028826678 | Reactome | ENSG00000151632; ENSG00000062282; ENSG00000106809; ENSG00000116133; ENSG00000196616; ENSG00000149485; ENSG00000004799; ENSG00000134824; ENSG00000139329; ENSG00000104812; ENSG00000278540; ENSG00000122176; ENSG00000186469; ENSG000001 | 1728; 2331; 4060; 1718; 2997; 31; 125; 5166; 4969; 1646; 54331; 9415; 84649; 3992 | 1481 | 1472 |

| | | | | | | | |
|---|---|---|---|---|---|---|---|
| | | | | 81019 | | | |
| Regulation of Complement cascade | 0.005308714 | 0.02895662 | Reactome | ENSG00000112936; ENSG00000039537 | 730; 729 | 24 | 24 |
| Propanoate metabolism - Homo sapiens (human) | 0.007186387 | 0.038045578 | KEGG | ENSG00000093144; ENSG00000278540 | 55862; 31 | 28 | 28 |
| Activation of Matrix Metalloproteinases | 0.008222042 | 0.042284788 | Reactome | ENSG00000143387; ENSG00000087245 | 1513; 4313 | 30 | 30 |
| Collagen degradation | 0.009893155 | 0.049465773 | Reactome | ENSG00000143387; ENSG00000087245 | 1513; 4313 | 33 | 33 |

Table S5: List of genes in the respective clusters from the overlap network, represented here in HGNC symbols.

| **Clusters** | **Genes** |
|---|---|
| 1 | ACACA, DHRS11, FADS1, FADS2, OLFM2, PDK4, SCD, SLC29A4 |
| 2 | CCND1, CTIF, DAB2IP, DGAT2, DTX1, FADS3, ITGA7, PLEKHG5, PMEPA1, RBPMS2, SYNPO, TENM4, TLCD2, TMEM120B, TMEM184B, TNFRSF25, ZNF219 |
| 3 | ABCC6, ADH1B, C6, C7, CAP2, ECHDC1, FAT1, GNG2, HSPA12A, KLB, KLHL31, MAL2, ME1, MRAP |
| 4 | GPLD1, KANK4, KIF1C, LEP, NPR3, NQO1, PDZD2, SLC24A3, SNCG, SRPX, VGLL3, VLDLR, VLDLR-AS1 |
| 5 | AKR1C2, DHCR24, FSTL3, LOXL2, TUBB2A |
| Un-clustered | AACS, CTSK, FFAR4, FMOD, GPNMB, GYS1, LUM, MMP2, OGN, SLC7A7 |
